# The prevalence of dyads in social life

**DOI:** 10.1371/journal.pone.0244188

**Published:** 2020-12-28

**Authors:** Leonard S. Peperkoorn, D. Vaughn Becker, Daniel Balliet, Simon Columbus, Catherine Molho, Paul A. M. Van Lange

**Affiliations:** 1 Department of Experimental and Applied Psychology, VU Amsterdam, Amsterdam, The Netherlands; 2 Human Systems Engineering, Arizona State University, Mesa, Arizona, United States of America; 3 Department of Psychology, University of Copenhagen, Copenhagen, Denmark; 4 Institute for Advanced Study in Toulouse, Toulouse, France; Goethe University Frankfurt am Main, GERMANY

## Abstract

A salient objective feature of the social environment in which people find themselves is group size. Knowledge of group size is highly relevant to behavioural scientists given that humans spend considerable time in social settings and the number of others influences much of human behaviour. What size of group do people actually look for and encounter in everyday life? Here we report four survey studies and one experience-sampling study (total *N* = 4,398) which provide evidence for the predominance of the dyad in daily life. Relative to larger group sizes, dyads are most common across a wide range of activities (e.g., conversations, projects, holidays, movies, sports, bars) obtained from three time moments (past activities, present, and future activities), sampling both mixed-sex and same-sex groups, with three different methodological approaches (retrospective reports, real-time data capture, and preference measures) in the United States and the Netherlands. We offer four mechanisms that may help explain this finding: *reciprocity*, *coordination*, *social exclusion*, and *reproduction*. The present findings advance our understanding of how individuals organize themselves in everyday life.

## Introduction

Our thoughts, emotions, and behaviours are influenced by those around us–in conversations at home, in shared activities such as going out for dinner with friends, and while working on projects with colleagues. In fact, humans spend considerable time in social settings [1]. Research finds that the *number of others* around us is key to understanding social phenomena such as free riding, trust, or helping behaviour. For example, people are less likely to free ride in smaller groups [2], more prone to trust others in smaller groups [3], and they are more inclined to feel responsible to help others in need when part of a small group [4], compared to larger group sizes.

Impressive strides have been made to expand theory on human group size, addressing interdependence at different levels of group size and activity (Caporael´s Core Configurations Model [5,6]) and how larger social group sizes may have selected for larger primate brains–Dunbar´s social brain hypothesis [7]. These theoretical frameworks focus on group sizes of about 2, 5, 30, 300 and network sizes of 5, 15, 50, 150, respectively. Despite the wide range in group sizes discussed in theoretical work, dyads have received the most research attention in practice.

Three examples illustrate this point. First, many economic games are two-player games or have only recently been extended to N-person games (e.g., the Prisoner’s Dilemma, Ultimatum Game, the Dictator Game, the Chicken Game, Trust Game; [8]). An Exception (more than two players) is, for instance, the Public Goods Game, but these are not as widely studied as the aforementioned two-player games. Second, social exchange has been primarily investigated as a dyadic phenomenon [9]. This is not surprising as social exchange involves reciprocity (“something has to be given and something returned”; p. 876). When exchange relations involve more people than two, typically it is no longer social exchange but economic exchange [10] in which a distribution rule or money plays an important role [11]. Third, in terms of early human development, major developmental theories focus on the dyad [12]. These include attachment theory, separation/individuation theory, and social learning theory. There is also a predominance of the dyad in friendship and romantic relationship research [13].

However, despite the fact that the dyad has received most research attention from scholars examining human interaction (e.g., game theory, social exchange, early human development, friendship, and romantic relationships) there is surprisingly little empirical research on what size of group people actually look for and encounter in everyday life. The focus on dyads in human interaction research is undoubtedly, in part, due to the methodological difficulties that arise when investigating larger group sizes, but it raises the question whether the dyad is also overrepresented in everyday life. Do we observe the dyad emerging as the predominant group size across a wide variety of social situations more than would be expected by chance?

We have noted that *systematic* research on group size is scarce, even though it appears an important variable in research on, for example, free riding [2] or helping behaviour [4]. Still, there have been incidental observations in older research that also support the primacy of the dyad. For instance, nearly seven decades ago, research found that the dyad constituted 71% of informal groups (e.g., shopping, swimming, stage plays) and work groups (e.g., construction, repair) [14,15]. Similarly, the dyad proved to be the most common group size at observations during free play at a nursery school, tourist parties visiting a national monument, and the size of dinner parties in a restaurant or conversation lounge [16,17]. More recently, a program of research by Dunbar and colleagues [18–21] has found support for Cohen’s [16] hypothesis that, for *conversation groups* specifically, there is an upper limit of four persons who can engage in active conversation, primarily due to cognitive constraints. It also seems plausible that conversations can be coordinated (e.g., turn-taking, being able to hear one another well) better within a group of up to four persons rather than groups much larger in size.

### The current research

This study addresses the question of whether we indeed observe the dyad emerging as the typical group size across a wide variety of social situations more than would be expected by chance. We provide preliminary empirical evidence from four survey studies (total *N* = 4124) that examined the size of the group in which people found themselves across social activities including dinner, movies/concert, off work chats, chats at work, projects, holidays, sports activity, and bars, as well as an experience sampling study (*N* = 274) of randomly selected situations people experience throughout daily life. Note that few scholars have tried to put forward situation taxonomies, and that these have not been successful in gaining strong support [22]. Therefore, we did not follow one particular situation taxonomy and instead our situations are eclectically derived from previous work. We define a group as “two or more persons who are interacting with one another in such a manner that each person influences and is influenced by each other person” [23] (p. 11). As such, when two friends go out to watch a football game, they are a group of two, within a larger aggregate of fans. In Study 1, we focused on Dutch participants’ past social activities. Study 2 replicated the results of the first study in a sample of US Americans, while Study 3 focused on US participants’ preferred group size for imagined future activities. In Study 4, same-sex social activities were assessed to examine sex differences in a US sample. The final experience-sampling study involved a real-time data capture approach using Dutch participants’ smartphones (Study 5).

## Results

### The dyad is frequently the most common group size

**Fig 1:**
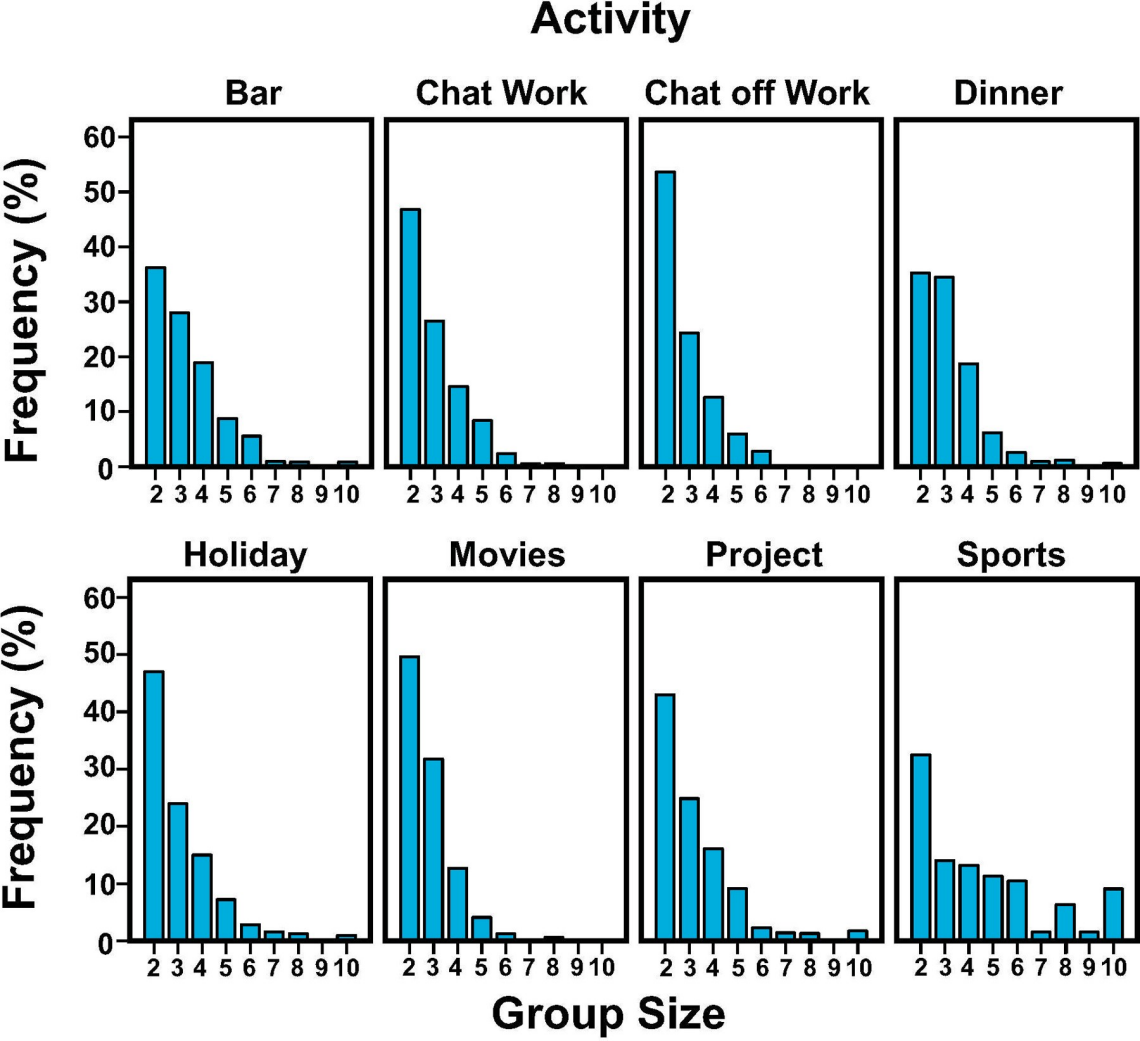
Frequency distributions of group size in eight daily activities sampled in Study 4.

Full information on data screening can be found in the Supporting Information (SI). We asked people to report the size of their social groups for a wide variety of activities. Group size frequencies are displayed in S1 Table and Figs 1 and S1–S3 in the main text. In all activities sampled, except for restaurant dinners, the dyad is the most common group size. This general pattern holds for Dutch participants in Study 1, American participants in Study 2, American participants reporting on *preferred* group size in Study 3, and for American participants reporting on activities involving *same-sex others* in Study 4. However, there are three cases where the dyad is the mode by only a small margin. These concern off-time chats in Study 1 (24.4% dyads, 24.3% groups of four), movies in Study 3 (34.6% for dyads and 30.8% for groups of four) and projects in Study 3 (29.6% for dyads, 25.9% for triads, and 25.5% for groups of four).

The typical group size for dinner parties was less clear. Future dinner parties of size 4 were strongly preferred in Study 3. Yet when people *actually* go out for dinner, they are typically with 2, 3, or 4 persons (Studies 1, 2, and 4). Similarly, participants in Study 3 preferred to go to the movies with two or four persons (34.6% vs. 30.8%), yet for *actual* movie visits, participants in Study 2 overwhelmingly chose to go as a dyad (51.7%) instead of as a group of four (15.5%). To a lesser extent this also holds true for chats off work and projects, as indicated in S2 and S3 Figs.

Moreover, as a general trend and perhaps not surprisingly, as group size increases the frequency of participants reporting to have been in such a group decreases. Except for sports activity, group sizes containing more than 10 individuals are rarely reported.

### The over-representation of dyads compared to other group sizes

The most commonly used count models are Poisson and negative binomial. If smaller groups are easier to coordinate, and coordination costs increase exponentially with group size, the observed data would best fit a Poisson distribution (shifted so that the minimum value is 2 rather than zero). In contrast, we found that it does not, having better overall model fit with zero-inflated Poisson distribution. This improvement in model fit using a zero-inflated rather than a standard Poisson distribution indicates that there were more dyads in the observed data than would be expected simply on the basis of ease of coordination. We tested the over-representation of dyads using zero-inflated Poisson and zero-inflated Negative Binomial regression. As noted above, we first reparametrized the outcome as the count of additional group members beyond the dyad. That is, by subtracting 2 from every observed group size (dyads would then obtain the value “0”), a count measure of group size greater than two resulted. If we observe an excess of dyads, the zero-inflated part of the model thus reflects dyad inflation. We first tested the fit of the Poisson model (with Participant sex as a predictor). We then fit the two zero-inflated models (i.e., zero-inflated Poisson and Negative Binomial, both of which accommodate overdispersion) and used a Vuong test to determine if they yielded better fit than the Poisson model. (A Vuong test is a test of the zero-inflated model against the noninflated model.) In almost every case they did (S2 Table, third column), indicating dyad-inflation.

The dyad-inflated models similarly support the notion that women are somewhat more dyadic—out of a total of 25 tests for all activities for four studies (5, 6, 6, and 8 activities in Studies 1 through 4, respectively), 13 Poisson regression tests show significant sex differences, and 12 of these effects survive the move to dyad-inflated models with at least *p* < .10 (see also S4 Fig). In no case do men show significantly more dyads or smaller group sizes relative to women. However, this also means that in about half of the activities, there were no significant sex differences and the differences we did find were generally small with some exceptions, such as same-sex sports, in which women were noticeably more dyadic than men (see S5–S8 Figs, for an overview of the sex differences by activity).

### Age and group size

Pearson correlations were performed to assess whether group size was associated with age. The correlations are reported in S3 Table. The significant associations were typically between small (.10) to medium (.30) in size. There was a positive correlation between age and dinner size for Dutch participants in Study 1, *r* = .11, *p* = .002. As age increases, so does the size of the dinner party. Yet, this result did not replicate with American participants in Studies 2–4. There was a negative correlation between age and group size at the movies for participants in Study 3 (*r* = -.08, *p* = .010) and men in Study 4 (*r* = -.15, *p* = .004). As age increases, group size at the movies decreases. Note, however, that this relationship was not found for participants in Studies 1–2, and for women in Study 4. For Dutch participants specifically, there was a positive correlation between age and conversation sizes (off work chats, *r* = .20, *p* < .001; chats at work, *r* = .18, *p* < .001). As age increases, conversation sizes become larger. There was also a positive correlation between age and project group sizes for participants in Study 1 (*r* = .21, *p* < .001), Study 2 (*r* = .07, *p* = .030), and Study 4 women (*r* = .11, *p* = .012) but not for participants in Study 3 and Study 4 men. Moreover, there was a negative correlation between age and holiday group size for participants in Study 2 (*r* = -.10, *p* = .003) but not for participants in Studies 3–4. Finally, Study 4 data indicated a negative relationship between age and sports group size in women (*r* = -.13, *p* = .009), but not men (*r* = -.02, *p* = .682); and a negative relationship between age and group size while visiting bars for men (*r* = -.14, *p* = .005), but not women (*r* = -.07, *p* = .109).

### Relationship status and group size

In order to assess the association between group size and relationship status (single versus in a relationship), Pearson correlations were performed and reported in S4 Table. For dinner parties there was a consistent positive relationship between group size and relationship status. Participants in a relationship reported larger dinner sizes than participants that were single. This holds true for Study 1 (*r* = .13, *p* < .001), Study 2 (*r* = .11, *p* < .001), Study 3 (*r* = .08, *p* = .013), Study 4 women (r = .09, p = .037) and Study 4 men (r = .16, p = .002). These effects can be considered small (that is, around .10). There were no significant correlations between group size and relationship status for movies, projects, and bars. For conversation sizes the pattern was less clear with only significant results for off work chats in Study 1 (*r* = .10, *p* = .003); chats at work in Study 2 (*r* = .09, *p* = .005) and Study 4 men (*r* = .14, *p* = .006). The positive sign indicates that participants in a relationship had larger conversation sizes. Finally, there was a significant negative correlation between group size and relationship status for Study 2 participants’ holidays (r = -.08, p = .013), indicating that participants in a relationship had smaller holiday groups; and a significant positive correlation for Study 4 men in the activity of sports (r = .10, p = .047), that is, men in a relationship engaged in sports activity with larger group sizes. The significant correlations for conversation sizes and holidays should be interpreted with caution as they are not consistently found across the studies reported here.

### Real-time experience-sampling data indicate that dyads predominate in daily life

Over the course of one week, participants (*N* = 274) were asked seven times a day to report the last situation they had experienced with another person since the last survey (i.e., situations were not sampled more than once). The experience sampling data yielded 10,933 total responses, 7,248 (66.29%) of which involved social situations. Forty-eight of these did not specify the number of other individuals present; therefore, for this analysis *N* = 7200 responses. The dyad was the most common group size (52.6%), followed by triads (18%), groups of four (9.1%), five (4.3%), six (2.5%), seven (1.6%), eight (0.9%), nine (1.0%), ten (0.5%), eleven (0.6%), and ‘more than 11’ (8.9%).

We again tested whether women encountered a greater proportion of dyadic situations than men. To do so, we fitted a logistic mixed effects model with random intercepts for participants and with gender as a level-2 predictor of the number of interaction partners in social interactions (7172 situations from 274 participants). Here, the outcome variable indicated whether the situation was dyadic (coded 1) or involving any other non-zero number of interaction partners (coded 0). We computed odds ratios for the fixed effects estimates. Analyses were conducted in R software version 3.4.4 using the tidyverse and lme4 packages. The hypothesis was not substantiated by the data. Women did not experience a greater proportion of dyadic (rather than non-dyadic) interactions than men, OR = 1.056, d = .03.

The detection of a sex difference in dyadic situations may be obscured by the overrepresentation of romantic couples for this unit size. Situations with romantic partners were indeed more dyadic than other situations, yet conducting the same analysis while excluding the situations with a romantic partner still found no significant sex difference (see S1 File).

## Discussion

The results of the present studies provide strong evidence for the prevalence of the dyad in daily life. Our data show that dyads are most common across a range of activities (e.g., conversations, projects, holidays, movies, sports, bars) obtained from three time moments (past activities, present, and future activities), sampling both mixed-sex and same-sex groups, with three different methodological approaches (retrospective reports, real-time data capture, and preference measures) in the United States and the Netherlands. With some exceptions, we also found similar patterns for men and women.

These results are in line with classic research conducted in the 1950s and 1970s [14–17] which also found dyads to be the most common group size at local sites with local participants (also see recent work by Dunbar and colleagues for conversation sizes specifically, [18–21]). Yet how do dyads relate to other, larger, group sizes? What theory can be used for understanding the prevalence of the dyad in daily life? To integrate the dyad with other group sizes, we briefly review Caporael’s Core Configuration Model [5,6] and although we did not test causal mechanisms in this study, we subsequently provide four possible reasons for the predominance of the dyad in everyday life.

### The core configurations model

Caporael [5,6] proposes that face-to-face groups are hierarchically structured in four core configurations. These represent kinds of interdependent *interactions* between people. The interdependency is determined by the type of task to be performed, the situated environment, and the physical attributes of the participants (i.e., “body morphology”). Group size is an important feature of the model but the core configuration sizes have little meaning without invoking the associated activities to be performed. The notion of being “core” configurations reflects the idea that these group configurations can be repeatedly observed in hunter-gatherer groups (and hence presumably across human evolutionary history), across an individual’s lifespan, and in daily life. These configurations occur for a specific purpose and the successful accomplishment thereof accounts for their continued recurrence. Moreover, it is proposed that cognitive processes have evolved and developed in the context of the core configurations and thereby also cause their recurrence over time.

The first core configuration is the dyad, an interaction between two entities (e.g., two humans, one human and an animal, or human-AI interaction). Tasks addressed by the dyad are, for example, internal fertilization in the context of sex, providing infant nutrition while the mother is breastfeeding the infant, or a child’s interaction with a peer or adult. One proposed function of the dyad is microcoordination (e.g., during facial imitation, gait adjustment between two people, or interactional synchrony during courtship initiation).

Second, the work group, family group, or team has a modal group size of 5 individuals and a range of 3–7 people. It refers to interactions in small face-to-face groups that have a common task orientation. Examples of small group tasks are foraging, hunting, and gathering. A modern world example would be the completion of an assignment by ad-hoc groups of students working together for brief periods in class (e.g., devising and assembling a basic technological device). The workgroup affords the function of distributed cognition across group members. This refers both to the sharing of cognitive resources (e.g., perception, knowledge, cues, focus of attention, inference, classification) in the pursuit of a shared representation of the task or problem and to a division of cognitive effort where there are specialties in the group over time allowing role-based trust.

Third, the deme, band, microband, or conceptual deme has a mode of 30 individuals with a group size range of 25–50 people. Note that Marilynn Brewer (a close collaborator of Caporael) proposed a group size range of 50–200 people for the deme/community in her 2015 keynote at the ICSD conference in Hong Kong, China [24]. The deme is similar in size to the extended family and modern-day classrooms. Common tasks of the deme are the movement from one location to another in hunter-gatherers, providing college students skill-based education (physical or cognitive), and the integration and redistribution of resources retrieved from smaller workgroups (e.g., meat from the hunt or research results from the lab). This configuration has the function of constructing a shared reality or worldview, a common bond identity and common knowledge. It also allows cooperative alliances to emerge which may lead to the breakaway of group members to form their own group in case of conflict or when the environment’s carrying capacity is reached.

Finally, the macrodeme or (seasonal) macroband has a modal group size of 300 individuals with a range of 100–500 individuals. Brewer [24] has instead provided a range of “300–1000 and beyond” people. The task of the macrodeme is the (seasonal, annual) gathering of people (bands of hunter-gatherers, scientists, businessmen) in the pursuit of exchanging resources, people, or information about more distant places or groups. The between-group mobility of people may involve mates in the case of hunter-gatherers or staff in the case of business or science. The function of the macrodeme has been proposed to be the stabilization and standardization of language. Stabilization indicating words referring to the same thing and standardization indicating that the members often use and understand the same words.

Whereas the dyad can rely on synchronization and reciprocity to obtain goals [25,26] and whereas workgroups can rely on mutual performance monitoring [27] and self-regulation [28] to achieve their objectives, members of the deme have to resort to other mechanisms to sustain cooperation and coordination. Several mechanisms have been proposed in the literature. These include informal sanctions [29]; indirect reciprocity [30]; and descriptive norms [31]. However, whereas in demes people are all individually known, this is not the case in macrodemes. As such the likelihood of knowing someone’s reputation may be insufficiently high for indirect reciprocity to serve as an effective mechanism to foster and maintain cooperation and coordination in very large groups [32]. In addition, it has been argued that there are mental constraints preventing people to keep track of the reputation of a large number of people at the same time [13,33]. Hence, other mechanisms such as proscriptive norms, institutions, and formal sanctions [34,35] may be necessary for people to work effectively together toward the accomplishment of their goals in macrodeme configurations.

### Four reasons for the prevalence of the dyad

More than larger sizes, dyadic interactions enable benefits from *direct reciprocity* [26]. In repeated dyadic interactions, the threat of non-reciprocation supports cooperative strategies such as tit-for-tat [26,36], but this mechanism breaks down in larger groups where freeriding is possible [37]. In a dyad, such strategies are efficient, as one’s choices are noticeably affecting one’s own and other’s outcomes. The partner may readily perceive that the other prefers stable cooperation over mutual selfishness, where exploitation would meet swift retaliation. However, even the small step from dyads to triads causes complexities in the workings (and effectiveness) of reciprocity, as one’s retaliatory action can no longer be delivered to the desired target only, the third person is equally affected [38]. When group size increases further, one is increasingly less able to induce cooperation in others through strategic signalling. One individual’s actions are both less likely to be perceived, and less likely to affect the others’ outcomes [37]. Thus, being part of a dyad (compared to larger sizes) allows for more control over the social situation toward the accomplishment of mutually beneficial outcomes.

Second, the detection of emotions and mental states via nonverbal cues is most likely to occur in dyads. Yet as group size increases, it becomes increasingly challenging to attend *n-1* communication channels in a group comprising *n* members [39]. Moreover, as group size increases, the number of interpersonal linkages along which *coordination* may be required increases sharply [39]. Indeed, coordination with multiple individuals is computationally complex and therefore individuals should prefer interaction partners with whom coordination is easier [40]. Coordination is more efficient with familiar others as learning about others’ preferences, intentions, and traits allows for improved behavioural anticipation [41]. As mentioned above, dyads are expected to be in a better position to attribute mental states (e.g., intentions, emotions, beliefs, desires, knowledge), to be clearer communicators, and hence to be behaviourally more predictable than larger groups. Thus, if people prefer those with predictable behaviours as interaction partners, such as familiar others [41], people may also have a preference for dyadic activities given their relatively predictable form and hence lower cognitive effort, compared with larger group sizes.

Third, group living provides various benefits. These include cooperation in the pursuit of difficult tasks, protection against danger, directing and receiving altruistic acts toward and from kin, the availability of allies, and high concentrations of mates [42]. However, group life also comes with liabilities, mostly in the form of conflict and competition. Examples include competition over material resources, high-status positions, or romantic partners. As such, individual competitors may want to exclude others who pose a threat to their interests (e.g., through derogating one’s competence and appearance or through spurious accusations [42]). Being excluded in an ancestral environment yielded dire prospects for survival and reproduction. Therefore, it is not surprising that (the threat of) social exclusion gives rise to anxiety [43] and social pain [44] and that people may be afraid to deviate from the group. Moreover, it has been argued that humans are highly sensitive to actual and threatened rejection and may possess an ostracism-detection system biased toward overdetection [45,46]. Whereas in larger groups social exclusion is a possibility, sometimes a threat, in a dyad one cannot exclude the other person without bearing the cost of becoming alone oneself. Seeing others together when one is alone may trigger affective distress, being a reminder of (the threat of) social exclusion [43] and as such is uncomfortable. Thus, even if one is generally embedded in a larger group and as such reaping its benefits, the dyad specifically may provide a relatively comfortable unit for social interaction in which self-monitoring and self-censorship can be somewhat relaxed, allowing for more authentic behaviour (i.e., in line with one’s own preferences and idiosyncrasies).

The final argument that may help explain the prevalence of the dyad in daily life concerns *reproduction* and infant-rearing activities, which take place mostly in pairs [5,6]. As mentioned above, this includes interactional synchrony during the initiation of courtship, which is a process confined to the mating couple [47]. For instance, it is difficult to conceive how three people would be able to nearly continuously look into each other’s eyes. Moreover, most people choose to engage in sexual activity with one other person during a sexual encounter. Next, the provision of infant nutrition through breastfeeding typically involves the mother and the infant in a breastfeeding dyad. Finally, alloparents (e.g., the grandmother [48], great-aunt or an older sibling [49]) can assist the parents in infant-rearing by watching over or feeding the child, allowing the parents to allocate their time in the pursuit of other activities. We argue that even with extensive alloparenting, the dyad may still be the most functional unit for interaction between caregiver and young child (e.g., allowing for facial imitation [5,6] and more efficient feeding).

Yet how do these theoretical reasons connect to the activities that were probed in the present studies? Our activities may have had low risk of exploitation, low costs in terms of commitment, and involved the exchange of resources likely of little consequence. However, the four proposed mechanisms we suggest are all attuned to the two-person interaction in daily life. This applies to *reciprocity* where the interactants engage in exchange and turn-taking. For instance, paying for the drinks at the movies, one may expect the other to pay next time. Reciprocity can happen “on the spot” (e.g., mutual self-disclosure), but more frequently unfolds in a step-by-step manner. As social transactions recur and gradually expand in significance, reciprocity results in fortified interdependent social bonds [10] (in the words of Allen et al. [50]: strong pair-wise ties). Besides trust, these processes are often accompanied by a series of emotions that may serve as internal pressures to maintain interpersonal cooperation: feelings of indebtedness, personal obligation, appreciation, and gratitude after receiving a favour [51]; feelings of guilt when failing to reciprocate [52]; anger when receiving substandard exchange [53], and forgiveness to pardon having been short-changed [54]. If the above line of reason is correct, it suggests the interesting hypothesis that various feelings and emotions such as indebtedness and gratitude are experienced most commonly and most strongly toward one other person instead of toward groups.

Second, in terms of *coordination*, in a dyadic interaction one enjoys relatively noise-free information. Compared to larger group sizes, dyadic interactions offer less room for hiding (e.g., question evasion, self-concealment [55]) and ambiguity, and this facilitates coordination. For instance, when a group of friends are planning a holiday trip, it may require significantly more effort to agree on a departure date and destination, not to mention all the small decisions to be made once there, compared with a dyad planning a similar trip together. Third, in a range of situations (e.g., holidays, projects, bars), being in a dyad may also entail being tied to another person, incentivizing both persons to be accommodating and cooperative to prevent *social exclusion* and having to fend for oneself in a sometimes daunting world. In a bar or club, for instance, it may feel uncomfortable to have no one to fall back on if needed. Finally, dyadic interaction also allows for relatively unconstrained flirting behaviour compared to situations where third-parties are present [56], reflecting the role of *reproduction* in the prevalence of the dyad in daily life. During a dyadic conversation, for example, a subtle courtship attempt (e.g., a wink or prolonged eye contact) can more freely be sent without potential interception costs from third parties. These mechanisms help to explain the primacy of the dyad because each of them supports cooperation in smaller groups–by which all individuals involved benefit. This is not to imply that cooperation does not occur in larger groups. We suggest that it is more challenging, however, and it involves qualitatively different mechanisms, such as indirect reciprocity [30] or third-party punishment [57]. In this empirical paper we echo the importance and prevalence of the (cooperative) dyad in society that has recently been demonstrated in mathematical and simulation analyses by Allen et al. [50].

### Sex differences

For our mixed-sex diary data (Study 5) we did not find significant sex differences. There were some significant differences for mixed-sex group activities sampled in Studies 1–3, but these were generally small. However, for *same-sex* groups in Study 4, the sex differences were somewhat more pronounced, where women are more dyadic than men. This applies for instance to sports (36.6% vs. 21.1%) or going to the movies (55.7% vs. 42.2%) and to a lesser extent going on holiday (49.9% vs. 43.1%) or out for dinner (37.8% vs. 32%). This latter finding is consistent with research on same sex friendship [58]. Using a sample of 112,000 Facebook profile pictures the authors found women to favour dyadic relationships, while men preferred larger male groups. Moreover, it has been demonstrated that men and women process social information differently in line with these differences in social structures, where women focus more on individuals while men focus more on groups [59,60]. Although the exact mechanism is unknown, various complementary explanations have been put forward to account for these sex differences (e.g., patrilocality [61]; by-product of pair-bonding [62]; maternal caregiving and empathic potential [63,64]).

### Limitations

Studies 1–4 have three limitations in common that should be taken into account. The situations that were sampled may not have been exhaustive or fully representative of daily life. Although we asked for the last time someone engaged in the activity, for some people it may have been weeks ago. Participants may also selectively remember an instance they particularly enjoyed. These concerns were addressed by Study 5, which used a real time approach in which the reference period is the present, or the last hours, and in which situations are sampled randomly. A limitation of Study 5, however, is that we did not code the situations that people reported. This is a fruitful avenue for future research given that situation taxonomies are currently lacking consensus and may be advanced by diary studies.

The current research focused on social activities and hence we have not probed individuals conducting activities alone [14,15]. Therefore, we do not know whether dyads would also be most frequent compared to individuals for various different tasks. In our studies, most participants engaged in activities with a few other people. However, there are certainly activities that require larger groups (e.g., barn-raisings). Future research could investigate whether even there, dyads may form the most effective subcomponents (“you hold the spike while I swing the hammer”). Indeed, given the aforementioned arguments (*reciprocity*, *coordination*, *social exclusion*, *reproduction*), these activities may also predominantly yield the dyad as the most common subunit in a larger collective. Related to this, because we focused on direct interaction, and used a definition of a group as “two or more persons who are interacting with one another in such a manner that each person influences and is influenced by each other person” [23], our results do not address the fact that groups can have psychological significance beyond direct interaction [65] and measuring groups differently may yield different results.

### Future research

One issue remaining for future research is to provide empirical evidence that the dyads that are so prevalent in various domains are also stable over time. Moreover, future research would do well to investigate the prevalence of the dyad in collectivistic daily life, to determine whether dyad inflation is generated by cultural factors as a necessary requirement for the effect to occur or whether an evolutionarily ingrained predisposition is sufficient in and of itself. If the former holds, then the effects should be observed only in individualistic countries, whereas if the latter holds, as argued by Caporael’s core configuration model [5,6], then the effects should be observable across individualistic and collectivistic cultures, and more generally, around the world.

## Materials and methods

### Study 1

All studies reported were approved by our faculty’s ethical review board (Vaste Commissie Wetenschap en Ethiek; VCWE). Participants indicated approval with our informed consent form which was displayed on a computer screen. In order to obtain a representative sample of the Dutch population we collected data through Flycatcher (www.flycatcher.eu), an online research company in the Netherlands. The Flycatcher panel consists of 16,000 participants who frequently participate in research. Participants receive points for every questionnaire that they complete. The accumulated points can subsequently be exchanged into gift vouchers.

#### Participants

A Dutch sample of 459 women (*M*_*age*_ = 50.04 years, *SD* = 16.12 years, range = 18–90 years) and 508 men (*M*_*age*_ = 51.66 years, *SD* = 15.57 years, range = 18–87 years) participated in this study and received points for their participation.

#### Measures

The respondents were presented with five daily activities and were asked to report the number of persons present in these activities while including themselves in the count. Participants read the following prompts. (1)*‘Think about the last time you went out for dinner with friends*.*’*, *(2)‘Think about the last time you visited a movie or concert with friends*.*’*, *(3)‘Think about the last time you had a conversation with others in your spare time*.*’*, *(4)‘Think about the last time you had a conversation with others at work*.*’*, *(5)‘Think about the last time you worked together on a common project or assignment as part of your work or training*.*’*, and then they were asked with how many persons, including themselves, did they engage in the respective activity. Note that the measures for this, and subsequent studies, are reported verbatim in the OSM.

#### Procedure

Participants filled out a large survey as part of a third wave of research on trust, relationships and moral dilemmas. The five questions on group size described above were part of this questionnaire and were added for exploratory purposes. After finishing all the questions, the respondents filled out their demographical information, were debriefed and thanked for their participation.

### Study 2 and 3

In Study 2 and 3 we aimed to replicate Study 1 while changing the population from Dutch to American participants and changing the data collection platform through which the responses were collected, from the Flycatcher Panel to an online crowdsourcing service: Amazon’s Mechanical Turk (https://www.mturk.com). In addition to the five activities sampled in Study 1, we added holidays as a sixth activity. In Study 2 respondents were asked to recall past activities in which they took part, while in Study 3 participants were asked to imagine themselves in a hypothetical future activity. The studies were run at the same time; participants were assigned to either Study 2 or to Study 3 after accepting our HIT (Human Intelligence Task) on M-Turk.

#### Study 2 Participants

We recruited 565 men (*M*_*age*_ = 30.85 years, *SD* = 9.59 years, range = 18–73 years) and 477 women (*M*_*age*_ = 34.92 years, *SD* = 12.15 years, range = 18–84 years) as participants in this study. The sample was restricted to respondents from the United States.

#### Study 3 Participants

The sample consisted of 591 men (*M*_*age*_ = 31.67 years, *SD* = 9.63 years, range = 16–75 years) and 470 women (*M*_*age*_ = 34.48 years, *SD* = 11.67 years, range = 19–76 years).

#### Measures

All participants were presented with six activities. Participants in Study 2 reported on past activities and were presented with the following six prompts: (1)*‘Recall the last time you went out for dinner with friends*.*’*, *(2)‘Recall the last time you went to the movies or a concert with friends*.*’*, *(3)‘Recall the last time you had a conversation with others in your spare time*.*’*, *(4)‘Recall the last time you had a conversation with others at work*.*’*, *(5)‘Recall the last time you worked together on a common project or assignment as part of your work or training*.*’*, *(6)‘Recall the last time you went on vacation*.’, and then were asked with how many persons, including themselves, did they partake in the respective activity. Below each of these questions the participant was presented with a text box that read ‘Number of persons:’ in which a number could be filled in. In contrast, participants in Study 3 reported on imagined future activities and were presented with the same six prompts as above except that the question wording was slightly different (e.g., ‘*Imagine that you go out for dinner with friends’; ‘Imagine that you go on vacation’)*. Next, participants were asked, with how many persons, including themselves, did they *prefer* to be in the respective activity. Underneath each question a textbox was presented that allowed the participants to fill in the number of persons. Finally, the following demographics were obtained from participants: *‘Do you identify as male or female’*, *‘What is your relationship status’*, *‘How long have you been together for’* (when respondents indicated being in a relationship), *‘What is your sexual orientation’*, *‘What is your age’*, *‘Are you Hispanic or Latino’*, *‘What best describes your ethnicity*?*’* and *‘Please enter your worker’s ID’*.

#### Procedure

Participants entered the experiment through clicking on our HIT on Amazon Mechanical Turk. The welcome screen presented the consent form and explained the study was about decision making in daily life. After providing consent, the various six questions were presented to the respondents and they filled out the number of persons that were present (Study 2) or preferred (Study 3) for each activity. Next, the participants reported their demographical information and were given a code that would allow respondents to obtain their reimbursement. Finally, participants were debriefed about the study.

### Study 4

In the previous studies we did not identify strong sex differences between men and women in the group sizes that were reported. It can be argued that no sex differences are expected in these situations as many or all participants may have reported on mixed-sex situations. Therefore, in Study 4 we aimed to focus on sex differences between men and women in the group sizes they report in various activities by asking explicitly about same-sex daily activities and by including two additional activities in which sex differences may be more likely: sports activity and visiting bars and clubs.

#### Participants

We recruited 450 men (*M*_*age*_ = 34.01 years, *SD* = 11.22 years, range = 18–75 years) and 604 women (*M*_*age*_ = 36.08 years, *SD* = 12.33 years, range = 18–84 years) as respondents in this study. The sample consisted of respondents from the United States. Participants received $0.40 for their contribution.

#### Measures

All participants were presented with eight same-sex daily activities that had happened in the past. The questions explicitly mentioned same-sex others in boldfaced text and clarified the sex of the others in parentheses. The following two example prompts were used for women (see SI for a complete overview of all questions).*‘Recall the last time you were physically engaged in a sports activity with* ***same sex others*** *(women only)*.*’*, *‘Recall the last time you were going out to a bar or club with* ***same sex others*** *(women only)*.*’*, and then were asked with how many persons, including themselves, did they engage in the respective activity. The questions for men were the same except for the information in parentheses, it specified ‘men only’. The demographical questions were identical to Studies 2 and 3.

#### Procedure

Participants accessed the study through Amazon Mechanical Turk. After reading the consent form the respondents proceeded with indicating their sex (male or female). Women were assigned to the female question format condition and men were assigned to the male format condition. Next, the participants indicated the group size that characterized the eight activities in which they had taken part in the past with same sex others. Finally, respondents provided demographical information, were provided the code to obtain payment and were debriefed about the study.

### Study 5

In Studies 1–4, we asked people to report on a wide variety of common situations. However, these categories may not have been exhaustive, and may not fully represent daily life. We therefore used experience sampling to obtain reports on random social situations. Moreover, in Studies 1, 2, and 4 we used retrospective reports to investigate group size. Although we asked for the *last time* someone engaged in a particular behaviour (e.g., going out for dinner), for some participants the last event may have been weeks ago. Finally, participants may remember a dinner that they especially enjoyed rather than the last dinner per se. To address these memory-related concerns we decided to use a real-time data capture approach in which the typical reference period is brief (i.e., the last hours, or even the present [66]).

#### Participants

We used data from the Interdependence in Daily Life Study [67]. A sample of 284 Dutch individuals (70% women) with a mean age of 35.55 years (*SD* = 16.02; range 18–80) were recruited through panel agencies and snowball sampling. The majority of the participants were born in the Netherlands (91.2%). Participants were paid €0.50 for every survey completed and obtained a bonus of €20 for a response rate of 80% or higher, as well as payments for other parts of the study not covered here, up to a total of €74.50.

#### Measures

Participants were asked whether they experienced a situation with another person since the last text message received. If so, respondents were asked with reference to the above situation: “*Apart from yourself*, *how many people were present*?” on a scale from 0 to 10 with the option to select ‘more than 10’.

#### Procedure

After an intake session during which demographics were recorded, as well as surveys and experimental tasks unrelated to this study, participants completed a seven-day-long experience sampling phase (10,933 responses; 78.6% response rate). Each day between 08:00 and 22:00, participants received seven messages at semi-random times (one message per two-hour time block, with at least 45 minutes in between). Participants received a survey link by text message sent through SurveySignal [68] and were prompted to recall the most recent situation they had experienced with another person (*k* = 7248 responses) since the last survey.

## Supporting information

S1 Fig Frequency distribution of group size in five daily activities sampled in Study 1. (EPS)

S2 Fig Frequency distribution of group size in six daily activities sampled in Study 2. (EPS)

S3 Fig Frequency distribution of group size in six imagined future daily activities sampled in Study 3. (EPS)

S4 Fig Mosaic plot indicating sex differences. (EPS)

S5 Fig Frequency distribution of group size in five daily activities indicating sex differences in Study 1. (EPS)

S6 Fig Frequency distribution of group size in six daily activities indicating sex differences in Study 2. (EPS)

S7 Fig Frequency distribution of group size in six imagined future daily activities indicating sex differences in Study 3. (EPS)

S8 Fig Frequency distribution of group size in eight daily same-sex activities indicating sex differences in Study 4. (EPS)

S1 Table Frequencies of group size in eight daily activities sampled across Studies 1–4. (PDF)

S2 Table Zero-inflated test results of poisson model and negative binomial model testing dyad inflation in Studies 1–4. (PDF)

S3 Table Summary of correlations between group size and age by activity for Studies 1–4. (PDF)

S4 Table Summary of correlations between group size and relationship status by activity Studies 1–4. (PDF)

S1 File Supplementary materials and methods. (PDF)
