# Supplementary material for: The prevalence of dyads in social life
Source: PLoS One. 2020 Dec 28;15(12):e0244188. doi: 10.1371/journal.pone.0244188 (PMC7769262; doi:10.1371/journal.pone.0244188)
Supplement: S1 Table — (PDF) [file pone.0244188.s009.pdf]

Table S1. Frequency Distribution of Group Size in Eight Daily Activities Sampled Across Studies 1–4

Size	Dinner					Movies					Off-Time Chat					Chat at Work				
	**S1**	**S2**	**S3**	**S4a**	**S4b**	S1	S2	S3	S4a	S4b	**S1**	**S2**	**S3**	**S4a**	**S4b**	S1	S2	S3	S4a	S4b
2	18.5	25.0	21.3	37.8	32.0	54.5	51.7	34.6	55.7	42.2	24.4	53.8	47.2	55.1	51.8	30.8	41.1	42.1	45.9	47.9
3	9.5	23.2	19.5	31.4	38.3	14.1	20.5	17.3	28.9	35.2	20.4	24.5	28.7	26.6	21.4	22.3	28.1	28.6	26.8	26.1
4	25.1	26.1	43.7	18.7	18.8	21.6	15.5	30.8	10.5	15.3	24.3	12.0	17.0	9.7	16.3	16.7	16.7	18.9	15.0	14.1
5	9.8	11.1	7.8	5.6	6.9	3.4	6.0	7.0	2.9	5.6	11.5	6.7	3.7	5.6	6.4	11.9	8.2	6.2	8.9	7.7
6	11.1	6.1	7.3	3.2	1.8	3.0	4.1	7.7	2.0	0.3	8.1	3.0	2.8	3.0	2.6	8.8	3.0	2.7	2.3	2.5
7	5.7	3.0	0.4	1.4	0.3	0.9	0.9	0.3	-	0.0	1.8	-	0.1	-	0.5	2.6	0.7	0.6	0.6	0.5
8	5.5	3.7	-	1.0	1.5	1.7	0.9	1.4	-	1.3	4.7	-	0.3	-	0.5	2.9	0.9	0.4	0.2	1.0
9	2.3	0.6	-	0.2	0.0	0.2	0.2	0.3	-	0.0	0.7	-	0.0	-	0.0	0.3	0.5	0.0	0.0	0.2
10	3.8	0.6	-	0.6	0.5	0.6	0.2	0.6	-	0.0	2.1	-	0.2	-	0.5	2.4	0.7	0.4	0.2	0.0
11	1.4	0.6	-	-	-	-	-	-	-	-	0.4	-	-	-	-	0.0	-	-	-	-
12	2.4	-	-	-	-	-	-	-	-	-	1.1	-	-	-	-	1.4	-	-	-	-
13	0.5	-	-	-	-	-	-	-	-	-	0.2	-	-	-	-	0.0	-	-	-	-
14	1.3	-	-	-	-	-	-	-	-	-	0.2	-	-	-	-	0.0	-	-	-	-
15	1.2	-	-	-	-	-	-	-	-	-	-	-	-	-	-	-	-	-	-	-
16	1.0	-	-	-	-	-	-	-	-	-	-	-	-	-	-	-	-	-	-	-
17	0.2	-	-	-	-	-	-	-	-	-	-	-	-	-	-	-	-	-	-	-
18	0.5	-	-	-	-	-	-	-	-	-	-	-	-	-	-	-	-	-	-	-
19	0.2	-	-	-	-	-	-	-	-	-	-	-	-	-	-	-	-	-	-	-

Size	Project					Holiday					Sports					Bar				
	**S1**	**S2**	**S3**	**S4a**	**S4b**	S1	S2	S3	S4a	S4b	**S1**	**S2**	**S3**	**S4a**	**S4b**	S1	S2	S3	S4a	S4b
2	33.2	32.5	29.6	44.4	41.1	-	39.5	42.3	49.9	43.1	-	-	-	36.6	21.1	-	-	-	37.0	35.4
3	18.0	23.0	25.9	23.3	26.9	-	16.6	12.8	24.0	23.9	-	-	-	11.8	13.3	-	-	-	28.0	28.0
4	14.1	19.9	25.5	17.1	14.7	-	18.9	23.6	13.7	16.8	-	-	-	11.8	11.8	-	-	-	17.3	20.8
5	8.3	10.4	11.6	8.7	9.6	-	10.0	8.3	6.6	8.1	-	-	-	8.4	11.8	-	-	-	10.0	7.1
6	8.5	6.0	4.8	1.7	3.0	-	6.1	7.3	2.9	2.7	-	-	-	6.3	12.5	-	-	-	4.9	6.3
7	3.2	1.8	0.3	1.3	1.5	-	2.4	0.6	1.2	2.0	-	-	-	2.2	0.5	-	-	-	1.3	0.5
8	5.1	2.7	1.3	1.3	1.3	-	2.6	3.1	0.7	2.0	-	-	-	5.1	6.3	-	-	-	0.2	1.6
9	0.5	0.4	0.0	0.2	0.3	-	0.9	0.1	0.2	0.0	-	-	-	1.2	1.5	-	-	-	0.0	0.0
10	3.7	1.8	1.0	1.9	1.5	-	1.5	1.9	0.7	1.3	-	-	-	7.0	9.3	-	-	-	1.3	0.3
11	0.7	0.1	-	-	-	-	0.5	-	-	-	-	-	-	0.7	2.8	-	-	-	-	-
12	1.7	0.8	-	-	-	-	0.4	-	-	-	-	-	-	2.7	3.3	-	-	-	-	-
13	0.5	0.1	-	-	-	-	0.5	-	-	-	-	-	-	0.7	0.3	-	-	-	-	-
14	0.0	0.3	-	-	-	-	0.1	-	-	-	-	-	-	0.2	1.3	-	-	-	-	-
15	1.7	-	-	-	-	-	-	-	-	-	-	-	-	1.9	0.5	-	-	-	-	-
16	0.5	-	-	-	-	-	-	-	-	-	-	-	-	0.5	0.5	-	-	-	-	-
17	0.0	-	-	-	-	-	-	-	-	-	-	-	-	0.2	0.0	-	-	-	-	-
18	0.2	-	-	-	-	-	-	-	-	-	-	-	-	0.0	1.0	-	-	-	-	-
19	-	-	-	-	-	-	-	-	-	-	-	-	-	0.2	0.0	-	-	-	-	-
20	-	-	-	-	-	-	-	-	-	-	-	-	-	1.9	2.0	-	-	-	-	-
21	-	-	-	-	-	-	-	-	-	-	-	-	-	0.0	0.3	-	-	-	-	-
22	-	-	-	-	-	-	-	-	-	-	-	-	-	0.5	0.3	-	-	-	-	-
23	-	-	-	-	-	-	-	-	-	-	-	-	-	-	0.0	-	-	-	-	-

Note. S1 = Study 1; S2 = Study 2; S3 = Study 3; S4 = Study 4. **Study 1**: Dinner: $N = 839$; Movies/Concert: $N = 644$; Off-time chat: $N = 843$; Chat at work: $N = 582$; Project: $N = 410$. **Study 2:** Dinner: $N = 976$; Movies/Concert: $N = 962$; Off-time chat: $N = 942$; Chat at work: $N = 968$; Project: $N = 978$; Holiday: $N = 924$. **Study 3**: Dinner: $N = 988$; Movies/Concert: $N = 975$; Off-time chat: $N = 967$; Chat at work: $N = 987$; Project: $N = 966$; Holiday: $N = 976$. **Study 4**: 'a' gives the frequency for women; 'b' gives the frequency for men. Dinner a: $N = 497$; Dinner b: $N = 394$; Movies/Concert a: $N = 456$; Movies/Concert b: $N = 372$; Off-time chat a: $N = 497$; Off-time chat b: $N = 392$; Chat at work a: $N = 514$; Chat at work b: $N = 403$; Project a: $N = 527$; Project b: $N = 394$; Holiday a: $N = 409$; Holiday b: $N = 297$; Sports a: $N = 415$; Sports b: $N = 399$; Bar a: $N = 468$; Bar b: $N = 379$.
