# Supplementary material for: The prevalence of dyads in social life
Source: PLoS One. 2020 Dec 28;15(12):e0244188. doi: 10.1371/journal.pone.0244188 (PMC7769262; doi:10.1371/journal.pone.0244188)
Supplement: S2 Table — (PDF) [file pone.0244188.s010.pdf]

Table S2. Zero-Inflated Test Results of Poisson Model and Negative Binomial Model Testing Dyad Inflation in Studies 1–4.

	Sex effect p-value (F<M) in Poisson Model	p-value for test of dyad inflation in Poisson Model	Vuong p-value for better fit of Inflated Model over Poisson	p-value for test of dyad inflation in Negative Binomial Model	Vuong p-value for better fit of Inflated Model over Negative Binomial
**Study 1**					
Dinner	.014*	<.001***	<.001***	<.001***	<.001***
Movie	.016*	.243	<.001***	.054	.087
Off-time Chat	.253	<.001***	<.001***	<.001***	<.001***
Chat at Work	.298	<.001***	<.001***	<.001***	<.001***
Project	.035*	<.001***	<.001***	.063	<.001***
**Study 2**					
Dinner	.602	<.001***	<.001***	<.001***	<.001***
Movie	.814	.002**	<.001***	.002**	.040*
Off-time Chat	.180	.003**	<.001***	.019*	.475
Chat at Work	.002**	<.001***	<.001***	-	-
Project	.504	<.001***	<.001***	.768	<.001***
Holiday	.001**	<.001***	<.001***	<.001***	<.001***
**Study 3**					
Dinner	.140	<.001***	.225	<.001***	.444
Movie	<.001***	<.001***	<.001***	<.001***	.328
Off-time Chat	<.001***	<.001***	.002**	.015*	.136
Chat at Work	.440	<.001***	<.001***	.063	.090
Project	<.001***	<.001***	.001**	<.001***	.091
Holiday	.023	<.001***	<.001***	<.001***	.014*
**Study 4**					
Dinner	.988	<.001***	.016*	.047*	.007**
Movie	<.001***	<.001***	.046	.031*	.084
Off-time Chat	.003**	<.001***	<.001***	.012*	.132
Chat at work	.865	<.001***	<.001***	.001**	.121
Project	.498	<.001***	<.001***	.018*	<.001***
Holiday	.002	<.001***	<.001***	.079	.003**
Sports	<.001***	<.001***	<.001***	<.001***	<.001***
Bar	.973	<.001***	<.001***	.013*	.020*

Note. * $p < .05$; ** $p < .01$; *** $p < .001$.
