# Supplementary material for: The prevalence of dyads in social life
Source: PLoS One. 2020 Dec 28;15(12):e0244188. doi: 10.1371/journal.pone.0244188 (PMC7769262; doi:10.1371/journal.pone.0244188)
Supplement: S3 Table — (PDF) [file pone.0244188.s011.pdf]

Table S3. Summary of correlations between group size and age by activity for Studies 1–4.

Activity	Study 1	Study 2	Study 3	Study 4 Women	Study 4 Men
Dinner	0.11**	<0.01	<0.01	-0.05	-0.10
Movies	0.02	-0.06	-0.08*	-0.02	-0.15**
Off Work Chats	0.20***	-0.03	-0.06	0.05	-0.07
Chats at Work	0.18***	0.05	-0.03	0.04	-0.03
Projects	0.21***	0.07*	0.02	0.11*	-0.02
Holidays	—	-0.10**	-0.06	-0.03	-0.07
Sports	—	—	—	-0.13**	-0.02
Bars	—	—	—	-0.07	-0.14**

Note. *p <.05; **p <.01; ***p < 0.001.
