# Supplementary material for: The prevalence of dyads in social life
Source: PLoS One. 2020 Dec 28;15(12):e0244188. doi: 10.1371/journal.pone.0244188 (PMC7769262; doi:10.1371/journal.pone.0244188)
Supplement: S4 Table — (PDF) [file pone.0244188.s012.pdf]

Table S4. Summary of correlations between group size and relationship status by activity Studies 1–4.

Activity	Study 1	Study 2	Study 3	Study 4 Women	Study 4 Men
Dinner	0.13***	0.11***	0.08*	0.09*	0.16**
Movies	0.05	-0.03	0.01	0.02	-0.01
Off Work Chats	0.10**	0.05	0.02	0.01	0.05
Chats at Work	0.06	0.09**	-0.02	-0.05	0.14**
Projects	-0.04	-0.04	-0.06	-0.04	0.03
Holidays	—	-0.08*	-0.02	0.04	0.08
Sports	—	—	—	<0.01	0.10*
Bars	—	—	—	0.06	0.07

Note. *p <.05; **p <.01; ***p < 0.001. Relationship status is coded as 0 = single; 1 = in a relationship.
