# Supplementary material for: The prevalence of dyads in social life
Source: PLoS One. 2020 Dec 28;15(12):e0244188. doi: 10.1371/journal.pone.0244188 (PMC7769262; doi:10.1371/journal.pone.0244188)
Supplement: S1 File — (PDF) [file pone.0244188.s013.pdf]

Supplementary Materials and Methods

Complete Question Wording Studies 1–4.

Study 1. The questions were the following. *'Think about the last time you went out for dinner with friends. With how many persons, including yourself, did you go out for dinner?'; 'Think about the last time you visited a movie or concert with friends. With how many persons, including yourself, did you visit the movie or concert?'; 'Think about the last time you had a conversation with others in your spare time. How many persons, including yourself, were involved in the conversation?'; 'Think about the last time you had a conversation with others at work. How many persons, including yourself, were involved in the conversation?'; 'Think about the last time you worked together on a common project or assignment as part of your work or training. With how many persons, including yourself, did you work together?'*

Study 2. Participants in Experiment 2 reported on past activities and were presented with the following six questions: *'Recall the last time you went out for dinner with friends. With how many persons, including yourself, did you go out for dinner?'; 'Recall the last time you went to the movies or a concert with friends. With how many persons, including yourself, did you go to the movies or concert?'; 'Recall the last time you had a conversation with others in your spare time. How many persons, including yourself, were involved in the conversation?'; 'Recall the last time you had a conversation with others at work. How many persons, including yourself, were involved in the conversation?'; 'Recall the last time you worked together on a common project or assignment as part of your work or training. With how many persons, including yourself, did you work together?'; 'Recall the last time you went on vacation. With how many persons, including yourself, did you go on vacation?'*

Study 3. Participants in Experiment 3 reported on imagined future activities and were presented with the following six questions: *'Imagine that you go out for dinner with friends.*

With how many persons, including yourself, do you prefer to go out for dinner?'; 'Imagine that you go to the movies or a concert with friends. With how many persons, including yourself, do you prefer to go to the movies or concert?'; 'Imagine that you have a conversation with others in your spare time. How many persons, including yourself, do you prefer to be involved in this conversation?'; 'Imagine that you have a conversation with others at work. How many persons, including yourself, do you prefer to be involved in this conversation?'; 'Imagine that you work together on a common project or assignment as part of your work or training. With how many persons, including yourself, do you prefer to work together?'; 'Imagine that you go on vacation. With how many persons, including yourself, do you prefer to go on vacation?

Study 4. The following questions were used for women. *'Recall the last time you went out for dinner with* ***same sex friends*** *(women only). With how many persons, including yourself, did you go out for dinner?'; 'Recall the last time you went to the movies or a concert with* ***same sex friends*** *(women only). With how many persons, including yourself, did you go to the movies or concert?'; 'Recall the last time you had a conversation with* ***same sex others*** *(women only) in your spare time. How many persons, including yourself, were involved in the conversation?'; 'Recall the last time you had a conversation with* ***same sex others*** *(women only) at work. How many persons, including yourself, were involved in the conversation?'; 'Recall the last time you worked together with* ***same sex others*** *(women only) on a common project or assignment as part of your work or training. With how many persons, including yourself, did you work together?'; 'Recall the last time you went on vacation with* ***same sex others*** *(women only). With how many persons, including yourself, did you go on vacation?'; 'Recall the last time you were physically engaged in a sports activity with* ***same sex others*** *(women only). With how many persons, including yourself, were you engaged in this sports activity?'; 'Recall the last time you were going out to a bar or club*

with ***same sex others*** *(women only). With how many persons, including yourself, did you go out with?'* The questions for men were the same except for the information in parentheses, it specified 'men only'.

Data Screening Studies 1–4.

Answers that indicated that the respondents had misunderstood the question or for whom the question did not apply (because they entered a group size value of zero or one) were dropped from the dataset. In most cases, this measure did not influence the total number of participants, as the participants would enter invalid responses for only one or two, but not all of the activities sampled. To assess whether there were any extreme outliers, we used the 3IQR outlier rule. That is, the quartile scores were obtained so as to calculate the interquartile range (Q3-Q1) and an outlier was taken to be any value beyond Q3 + 3*IQR.

Study 1

The data screening measure of dropping values of zero and one did not affect the total number of participants for Study 1 ($N = 968$). Note that one person was dropped from the data set as the participant indicated being male in 2012 and female in 2014. Therefore, the total number of participants is $N = 967$ ($M = 50.89$ years, $SD = 15.85$), as reported in the main text.

Study 2

Of the 1,076 participants, only data for those providing complete responses (N = 1,042; $M = 32.71$ years, $SD = 11.02$) were analysed. Complete responses were taken to be those where the participant had filled out the last question with a valid M-Turk ID. Similar to the procedure in Study 1, we dropped responses that entered values of zero or one. Finally, we used the 3IQR outlier rule to identify and exclude outliers for the various different activities included in this study.

Study 3

All complete responses were retained for analysis (N = 1,061; M = 32.91 years, SD = 10.67), whereas incomplete responses were excluded (N = 19). The other screening measures (i.e., the removal of zeros and ones entered as group sizes, and the outlier rule) were identical to Study 2.

Study 4

Out of the original 1,108 respondents, 54 participants were excluded from the analyses due to incomplete responses. Therefore, for this analysis, sample size is N = 1,054 (M = 35.20 years, SD = 11.90). The same data screening measures were taken as described in Studies 2 and 3.

Overrepresentation of Romantic Partners in Study 5

Situations involving people's romantic partners may have been overrepresented in Study 5. These situations made up 18.5% of all reported situations, and were highly dyadic (79.6%). We therefore re-ran the analysis of gender differences excluding these situations. However, the effect of gender on the prevalence of dyads remained small and non-significant (OR = 1.11, Z = .721, p = .471). Note below that situations with romantic partners were more dyadic than other situations:

Partner: 79.6%

Family: 48.5%

Friend(s): 52.0%

Colleagues/Classmates: 41.3%

Supervisors/Instructors: 41.1%

Strangers: 41.2%

Acquaintances: 48.0%

Others: 44.8%
